# The study of two barley *Type I-like MADS-box* genes as potential targets of epigenetic regulation during seed development

**DOI:** 10.1186/1471-2229-12-166

**Published:** 2012-09-17

**Authors:** Aliki Kapazoglou, Cawas Engineer, Vicky Drosou, Chrysanthi Kalloniati, Eleni Tani, Aphrodite Tsaballa, Evangelia D Kouri, Ioannis Ganopoulos, Emmanouil Flemetakis, Athanasios S Tsaftaris

**Affiliations:** 1 Institute of Agrobiotechnology (INA), CERTH, Thermi-Thessaloniki, GR-57001, Greece; 2 Department of Genetics and Plant Breeding, Aristotle University of Thessaloniki, Thessaloniki, GR-54124, Greece; 3 Department of Agricultural Biotechnology, Agricultural University of Athens, Iera Odos 75, Athens, GR-11855, Greece

**Keywords:** MADS-Box, Epigenetic regulation, Chromatin, DNA methylation, Histone methylation, Seed development, Endosperm, Retrotransposon, Barley

## Abstract

**Background:**

MADS-box genes constitute a large family of transcription factors functioning as key regulators of many processes during plant vegetative and reproductive development. *Type II MADS-box* genes have been intensively investigated and are mostly involved in vegetative and flowering development. A growing number of studies of *Type I MADS-box* genes in Arabidopsis, have assigned crucial roles for these genes in gamete and seed development and have demonstrated that a number of *Type I MADS-box* genes are epigenetically regulated by DNA methylation and histone modifications. However, reports on agronomically important cereals such as barley and wheat are scarce.

**Results:**

Here we report the identification and characterization of two *Type I-like MADS-box* genes, from barley (*Hordeum vulgare*), a monocot cereal crop of high agronomic importance. Protein sequence and phylogenetic analysis showed that the putative proteins are related to Type I MADS-box proteins, and classified them in a distinct cereal clade. Significant differences in gene expression among seed developmental stages and between barley cultivars with varying seed size were revealed for both genes. One of these genes was shown to be induced by the seed development- and stress-related hormones ABA and JA whereas *in situ* hybridizations localized the other gene to specific endosperm sub-compartments. The genomic organization of the latter has high conservation with the cereal *Type I-like MADS-box* homologues and the chromosomal position of both genes is close to markers associated with seed quality traits. DNA methylation differences are present in the upstream and downstream regulatory regions of the barley *Type I-like MADS-box* genes in two different developmental stages and in response to ABA treatment which may be associated with gene expression differences.

**Conclusions:**

Two barley MADS-box genes were studied that are related to *Type I MADS-box* genes. Differential expression in different seed developmental stages as well as in barley cultivars with different seed size was evidenced for both genes. The two barley *Type I MADS-box* genes were found to be induced by ABA and JA. DNA methylation differences in different seed developmental stages and after exogenous application of ABA is suggestive of epigenetic regulation of gene expression. The study of barley *Type I-like MADS-box* genes extends our investigations of gene regulation during endosperm and seed development in a monocot crop like barley.

## Background

In angiosperms, the endosperm of the developing seeds is formed as a result of the double fertilization event. Fertilization of the egg cell by a sperm cell from the male gametophyte generates the diploid embryo from which the tissues, organs, and shoot meristems of the plant will be generated. Fertilization of the adjacent central cell by a second sperm cell forms a triploid endosperm which supports embryo growth and development by producing storage proteins, lipids and starch [1,2]. During this process, a large number of genes are activated. Epigenetic regulatory considerations become important in relation to which parental allele will be expressed and in which reproductive tissue, a factor that ultimately governs among other things the size of the endosperm [3-6].

Developmental transition of plants from the vegetative to the reproductive stage and from the floral stage to the seed stage relies on the activity of MADS-box transcription factors. With the completion of the Arabidopsis genome, more than 100 genes encoding for MADS-box transcription factors were uncovered which can be phylogenetically classified in five clades, termed MIKC, Mα, Mβ, Mγ and Mδ (Arabidopsis Genome Initiative, 2000) [7,8]. The Mα, Mβ, Mγ subfamilies comprise the Type I lineage, whereas the MIKC and Mδ constitute the Type II lineage. Similarly, phylogenetic analysis based on the completed rice genome revealed 44 *Type II MADS-box* genes, of the MIKC and Mδ clades, and 35 *Type I MADS-box* genes of the Mα, Mβ, Mγ clades [9]. The two lineages are proposed to have arisen by a gene duplication that took place in the common eukaryotic ancestor more than a billion years ago [10-13]. *MIKC* genes harbor the characteristic MADS-box domain (M), the Intervening domain (I), the Keratin-like domain (K) and the C-terminal domain (C). In contrast, the *Type I* genes contain only the MADS-box domain (M). Although the *Type I* genes represent about 60% of the total *MADS* genes in Arabidopsis, they have only recently started to be studied, whereas the *Type II* genes have been extensively investigated both structurally and functionally [8,14-17]. This is due mostly to the fact that *Type I MADS* genes function during early gametogenesis, embryogenesis and seed formation and consequently homozygous mutants are lethal. In addition their expression is very low during these developmental stages [8,18]. Among the few *Type I* family members that have been studied is *PHERES 1* (*PHE1*) [also known as *AGAMOUS-LIKE37* (*AGL37*)], a *Mγ-type* gene with an important role in Arabidopsis gamete and seed development [19]. Epigenetic regulatory mechanisms have been implicated in the transcriptional control of this gene. The *PHE1* gene is expressed transiently at high levels immediately after fertilization in a parentally imprinted manner where the paternal allele is expressed whereas the maternal allele is silenced [19,20]. Both DNA and histone methylation are responsible for these silencing events [19-21]. The PRC2 Polycomb group complex is partially responsible for keeping the maternal allele silent in the female gametophyte and in the seed after fertilization [19-21]. This is achieved through the histone methylation activity of the PRC2 complex, conferred by one of its subunits, the histone methyltransferase MEDEA (MEA) with histone 3 lysine 27 trimethylation activity (H3K27me3). The PRC2 complex also restricts the expression of *PHE1* to the chalazal domain of the endosperm after fertilization. Arabidopsis *mea* mutants show upregulated *PHE1* expression and form defective seed-like structures before fertilization and endosperm overproliferation after fertilization [19,20,22]. Furthermore, a distantly located region downstream of paternal *PHE1* was found to have a DNA methylation requirement for *PHE1* expression [22].

Additional *Type I MADS-box* genes have been implicated in gamete and seed development. The *AGAMOUS-LIKE23 (AGL23)* gene was found to regulate female gametophyte formation and normal embryo development [23]. *AGAMOUS-LIKE 80* (*AGL80*) was shown to be critical for central cell and endosperm development in Arabidopsis [24]. In *agl80* mutants the formation of the central cell is defective, and endosperm development fails to initiate after fertilization. Similarly, *AGAMOUS-LIKE 61* (*AGL61*) also termed *DIANA*, plays an important role in central cell and endosperm formation, in Arabidopsis [16,25]. Like *agl80*, *agl61* mutants have aberrant central cell morphology which degenerates before fertilization occurs, and endosperm development does not take place post fertilization [16,25]. Both genes are expressed exclusively in the central cell and early endosperm and this along with their similar mutant phenotype suggests that AGL61 and AGL80 proteins may function as heterodimers within the central cell. In agreement to this, AGL61 and AGL80 proteins were found to interact in yeast two-hybrid assays [16,25]. Of equal interest is *AGL62* with high expression in the early nuclear endosperm and sharp decline right before cellularization [26]. In *agl62* mutants, the endosperm cellularizes prematurely indicating that the *AGL62* is required for repression of precocious cellularization during the syncytial phase*. AGL62* is under the epigenetic control of *PRC2* genes, as in *prc2* mutants *AGL62* fails to become silent and endosperm cellularization is arrested [26]. Thus, *AGL62* seems to regulate the timing of endosperm cellularization, which is triggered epigenetically by PRC2-mediated *AGL62* silencing. Likewise, a recent study on *AGL36* demonstrated that the expression of the *AGL36* maternal allele is epigenetically controlled in a sequential manner, firstly upregulated by the function of DEMETER (DME), a DNA glycosylase enzyme responsible for demethylating *MEDEA*, and then downregulated during endosperm development by the PRC2 complex [27]. Understanding the genetic and epigenetic processes controlling the timing of endosperm cellularization could be of particular interest in agriculture, as premature or delayed cellularization is associated with small and large seed size and weight, respectively.

An extensive genome-wide study of 60 *Type I MADS-box* genes in Arabidopsis uncovered a cell-type-specific expression pattern during female gametophyte and early endosperm development [18]. Most genes are expressed in the central cell and antipodal cells in the female gametophyte and in the chalazal and peripheral endosperm of 1–2 days after fertilization (DAF) developing seeds. These data are in agreement with the results from earlier functional studies of individual genes like *PHE1, AGL23*, *AGL61, AGL62* and *AGL80* and propose a role for *Type I MADS-box* genes in female gametophyte and endosperm development.

Considerably less is known about *Type I MADS-box* genes in other plant species, especially monocots. In wheat, 42 *MADS-box* genes were identified *in silico,* of which 8 were classified as *Type I*[28]. In rice, a global-scale microarray expression analysis identified an expression pattern for all *Type II* and *Type I MADS-box* genes during vegetative and reproductive development [9]. Overall *Type I MADS-box* genes had lower expression levels than *Type II MADS-box* genes. Certain *Type I* genes were expressed throughout development, whereas others exhibited more specific expression in particular tissues. For example, four rice *Type I* genes, of the subclass Mα, were found predominately expressed in seeds 5–20 days after fertilization suggesting a role for these genes in seed development. Recently, a rice *Type I MADS-box* gene, *OsMADS87,* was reported to be maternally expressed in rice endosperm and associated to endosperm developmental transitions caused by interspecific hybridization [29].

Cereal crops account for about 50% of global human calorific intake with the endosperm of cereal seeds being one of the most important sources (faostat.fao.org). Chief among these monocots are barley, wheat, rice and maize. Besides human nutrition, cereal crops also represent major sources of feedstock, fiber and recently biofuel substrates. Contrary to Arabidopsis and other dicots where the endosperm is consumed during seed development, in monocots, such as barley and other cereals, the endosperm persists and constitutes the nutritional part of the seed containing storage proteins and starch.

Considering the implications of *Type I MADS-box* genes on seed development and the agronomic importance of cereal endosperm, we set out to identify and characterize *Type I MADS-box* genes in barley. At least three of the Arabidopsis *Type I MADS-box* genes, *PHE1, AGL62*, and *AGL36* are under epigenetic regulation mediated, in part, by the chromatin repressive enzymatic complex PRC2. Our group has recently characterized barley genes encoding a putative PRC2 Polycomb group complex [30]. To further focus our research efforts in this area and study potential barley targets of a PRC2 complex, we report here the identification and structural characterization of two barley *Type I-like MADS-box* genes. Their expression has been studied in different tissues and seed developmental stages, in two cultivars with varying seed size, and after exogenous application of the developmental- and stress-related phytohormone ABA. In addition, their genomic organization was examined and compared to their cereal homologues. Finally, the 5’upstream regions were analyzed for conserved *cis* regulatory elements and the DNA methylation patterns of upstream and downstream regions were investigated in two tissues with differential *Type I-like MADS-box* gene expression.

## Methods

### Plant material

Commercial barley cultivars, Caresse, Byzantio, and Ippolytos differing in seed size and weight were planted in the field and were the source of total RNA for expression analysis. For Caresse, the weight of 1000 grains is 50–55 gr, and 98% of seeds have diameter longer than 2.5 mm, for Byzantio the weight of 1000 grains is 36–42 gr and 75% of seeds have diameter longer than 2.5 mm, whereas for Ippolytos, seeds weight 25–31 gr per 1000 grains and only 35–45% of seeds have diameter longer than 2.5 mm (http://www.cerealinstitute.gr).

### Hormonal treatment

Seven-day-old seedlings (Caresse) grown in a growth chamber (16 hours (h) light, 8 h darkness, at 22°C) were sprayed with 100 μM ABA, (abscisic acid +/− *cis*, *trans*-ABA, SIGMA) and 100 μM JA (methyl jasmonate, ALDRICH). Aerial parts of plants were collected at 6 h and 24 h after treatment and immediately stored in liquid nitrogen. Aerial parts from five plants were pooled together for RNA extraction for each time point. Control plants were sprayed with water plus 0.2% Tween.

### RNA isolation and first strand cDNA synthesis

Total RNA was isolated from roots, shoots, apical meristems, first leaves of seedlings, flowers before fertilization (immature flowers), seeds 1–3, 3–5, 5–10, 10–15, 15–20 days after fertilization (DAF), and aerial parts after hormonal treatments, respectively, using TRI REAGENT (SIGMA) according to the instructions of the manufacturer. First strand cDNA synthesis was performed using 1.0 μg total RNA, 0.5 μg 3’ RACE Adapter primer, 5’-GGCCACGCGTCGACTAGTAC (T)_17_-3’ (Invitrogen), 1 mM dNTPs and 200U of Superscript II (Invitrogen) in 20 μL total volume, according to the specifications of the manufacturer.

### Protein sequence analysis

1895 viridiplantae proteins sequences from UniProt with a statistically significant hit for the MADS-box domain (PF00319: SRF-type transcription factor (DNA-binding and dimerization domain) were collected from the Pfam database [31]. After sequence fragments were filtered out, the resulting set was the subject of an all-against-all similarity detection step using the BLAST algorithm. We used the bidirectional best hit approach [32] for discovering putative orthologous proteins; we also asked for any orthologous sequence to be one of at least twenty sequences with the MADS-box domain from the corresponding species, so that we reduce the chance of best hits due to lack of sequence context. The multiple alignment was created with MAFFT [33] and edited with JalView version 2.5 [34] after which the phylogenetic tree was constructed in the same software with the Neighbor Joining method using a BLOSUM62-based distance measure. The phylogenetic tree was calculated using MEGA 3.1 software [35] by the Neighbor-Joining Method with p-distance correction [36]. Bootstrap values were obtained from 1000 bootstrap replicates. Accession numbers for sequences used for alignments and phylogenetic analysis are indicated in Table 1.

**Table 1:** Type I MADS-box sequences used for alignments and phylogenetic tree construction

**Organism**	**Gene name**	**Accession number**	**Type I**
			**Cereal type-I-like**
Hordeum vulgare	HvOS2	HM130526.1 TC178280	
Triticum aestivum	TaAGL33	ABF57950.1 Q1G159	
Brachypodium distachyon	Bradi2g59190	Bradi2g59190	
Hordeum vulgare	HvOS1	HM130525.1 BG365393	
Triticum aestivum	TaAGL42	ABF57942.1 Q1G167	
Brachypodium distachyon	Bradi2g59120	Bradi2g59120	
Zea mays	ZmB4FML1	NP_001140218.1	
Orysa sativa	OsMADS65	Os01g0922800 Q9XJ61	
			**Mα**
Arabidopsis thaliana	AtAGL62	NP_200852.1 Q9FKK2	
Arabidopsis thaliana	AtAGL61	NP_850058.1 A5AZX6	
Arabidopsis thaliana	AtAGL23	NP_176715.1	
Arabidopsis thaliana	AtAGL28	NP_171660.1	
Poplar trichocarpa	AGL62-like	XP_002313197.1 B9HN34	
Vitis vinifera	AGL62-like	A5AZX6	
Ricinus communis	AGL62-like	B9S7W9	
Orysa sativa	OsMADS71	Os06g22760.1	
Orysa sativa	OsMADS78	Os09g02830.1	
Orysa sativa	OsMADS79	Os01g74440.1	
			**Mβ**
Arabidopsis thaliana	AtAGL47	NP_200380.1	
Arabidopsis thaliana	AtAGL82	NP_200697.1 Q9FIM0	
Poplar trichocarpa	PtMADS-I-like	XP_002301840	
Orysa sativa	OsMADS90	Os07g04170	
Orysa sativa	OsMADS91	Os01g11510.1	
Orysa sativa	OsMADS96	OsO1g67890	
			**Mγ**
Arabidopsis thaliana	PHE1 AGL37	NP_176712.1 O80805	
Arabidopsis thaliana	PHE2 AGL38	NP_176709.2 Q7XJK8	
Arabidopsis thaliana	AGL80	NP_199678.1 Q9FJK3	
Orysa sativa indica	OsAGL80-like	EAY73584.1 Q75IC5	
Orysa sativa japonica	OsjAGL35-related	BAD81343.1	
Orysa sativa	OsMADS82	Os04g24800	
Orysa sativa	OsMADS83	Os04g24810	
Orysa sativa	OsMADS85	Os04g25920	
Poplar trichocarpa	PtAGL80-like	XP_002329991.1 B9I7J4	
Petunia hybrid	PhAGL80-like	B6DT62	
Ricinus communis	RcAGL80-like	B9S273	
Vitis vinifera	VvAGL80-like	A5BJU9	

### Genomic organization

The genomic sequences of *Brachypodium distachyon* _*Bradi2g59120*, *Brachypodium distachyon_ Bradi2g59190*, *Oryza sativa*_*OsMADS65*_Q9XJ61 (Os01g0922800), and *Zea mays*_*ZmMADS1*_GRMZM2G171650_B4FML1, were downloaded from the Phytozome database (http://www.phytozome.net/). The sequences of the two full length barley *Type-like MADS-box* ESTs were used to interrogate the barley database http://webblast.ipk-gatersleben.de/barley/index.php for detection of genomic sequences. Genomic organization of exons and introns was obtained using the mRNA-to-genomic alignment Spidey tool, in NCBI (http://www.ncbi.nlm.nih.gov/spidey). Detection of retroelements was performed with the MASiVE and LTRharvester tools (http://tools.bat.ina.certh.gr/masive/) (http://tools.bat.ina.certh.gr/ltrharvester/) and homology was visualized with Circoletto (http://tools.bat.ina.certh.gr/circoletto/), all three tools developed in-house at INA, by the Bioinformatics Analysis Team (BAT).

### Mapping *in silico*

An *in silico* visual comparative analysis was performed against the Barley, OPA 2009, Consensus-Hordeum-OPA-2009-3 H [37], the Barley, OPA123-2008, Consensus-Hordeum-OPA123-2008-3 H and the Barley, OWB, OPA2008-Hordeum-OWB-OPA2008-3 H [38] using the HarvEST tool and the comparative map viewer (Cmap) available at Gramene (http://www.gramene.org/cmap/).

### Expression analysis of barley Type I-like MADS-box genes

Qualitative RT-PCR and quantitative real-time RT-PCR was performed with cDNA synthesized from 1 μg of total RNA from roots, stems, meristems, leaves, immature flowers, seeds 1–3 DAF, 3–5 DAF, 5–10 DAF, 10–15 DAF and 15–20 DAF and aerial parts of seedlings after ABA and JA treatment. For real-time PCR, each sample reaction was set up in a PCR reaction mix (20 μl) containing 5 μl of the 1:50 diluted cDNA, 0.25 μM of each primer and 1X Platinum SYBR Green qPCR Supermix-UDG (Invitrogen, Paisley, UK) and using the Corbett Rotor Gene 6000. Each reaction was performed in triplicates. General thermocycler conditions were 50°C for 2 min, 95°C for 2 min, then 42 cycles of 95°C for 15 sec, annealing [53°C for *HvOS1* and 57°C for *HvOS2* for 20 sec, extension 72°C for 20 sec, then 72°C for 5 min. To identify the PCR products a melting curve was performed from 65°C to 95°C with observations every 0.2°C and a 10-s hold between observations. Relative quantification was performed using actin as the reference gene and *HvActinF / HvActinR* as primers. The barley genes *HVA22* and *HvADC* (arginine decarboxylase 2) which are known to be induced by ABA and JA, respectively [39,40], were used as positive controls. All primers used in expression analysis correspond to non-conserved regions and are shown in (Additional file 1: Table S2).

### *In situ* hybridization

In situ hybridization experiments were performed as it has been described previously [41]. Briefly, seeds were fixed in 4% (w/v) paraformaldehyde supplemented with 0.25% (v/v) glutaraldehyde in 10 mM sodium phosphate buffer (pH 7.4) for 4 h in a vacuum aspirator. Fixed tissues were block-stained in 0.5% (w/v) safranin, dehydrated through ethanol series, embedded in paraffin and cut into 8 mm-thin sections. Antisense RNA probes labelled with digoxigenin-11-rUTP (Boehringer Mannheim, Mannheim, Germany) were originated from PCR-generated templates incorporating T3 polymerase sites. The probe was designed close to the 3’-UTR of the gene and its length was 202 bp. Sections were prepared for hybridization as described before [42] and hybridized overnight at 42°C in 50% (v/v) formamide, 300 mM NaCl, 10 mM Tris–HCl pH 7.5, 1 mM EDTA, 0.02% (w/v) Ficoll, 0.02% (w/v) polyvinylpyrrolidone, 0.025% (w/v) bovine serum albumin (BSA), 10% (v/v) dextran sulfate and 60 mM DTT. After hybridization, the sections were treated with a solution containing 500 mM NaCl, 1 mM EDTA, 10 mM Tris–HCl and 50 μg/ml RNase A. Finally, sections were washed several times in a 2xSSC solution. Hybridization signals were visualized with anti-digoxigenin antibodies conjugated with alkaline phosphatase. Images were processed using Photoshop 7 software (Adobe Systems Inc., San Jose, CA, USA).

### Genome walking and 5’ upstream sequence analysis

Genome walking experiments were conducted on leaf genomic DNA obtained from the barley Caresse cultivar. The Clontech genome walking kit was used and procedures were conducted according to manufacturer’s specifications. Sequence information was obtained by doing three progressive rounds of genome walking using EST sequence data as starting points. Statistically significant prediction for CpG islands was performed using the online predictor, which is part of the sequence manipulation suite at http://www.bioinformatics.org/SMS/index.html. The prediction of the putative *cis* acting elements was accomplished using the TSSP /Prediction of PLANT Promoters algorithm (Using RegSite Plant DB, Softberry Inc.) in the SoftBerry database (http://linux1.softberry.com/cgi-bin/programs/promoter/tssp.pl) and PlantCARE (http://bioinformatics.psb.ugent.be/webtools/plantcare/html/). The position of the putative 5’ upstream regulatory elements is indicated according to the start codon (ATG).

### DNA methylation assays

Genomic DNA was prepared from immature flowers and from 1–3 DAF seeds (Caresse) with Qiagen columns following the protocol of the manufacturer (Qiagen Plant genomic DNA kit). Cytosine DNA methylation was analyzed by restricting 1 μg of genomic DNA from each sample with the methylation-dependent enzyme *Mcr*BC (NEB Biolabs), according to the manufacturer’s instructions, and PCR-amplifying equal quantities of *Mcr*BC-treated and untreated samples. Primers used are shown in (Additional file 1: Table S2).

## Results

### Identification of Type I-like MADS-box genes in barley and protein sequence analyses

Initial efforts to identify orthologues of *Type I MADS-box* genes in barley EST databases via BLAST and other sequence similarity-based approaches failed to result in significant hits. Additional efforts using degenerate primers designed using Arabidopsis sequence data and barley cDNA from multiple cultivars did not result in target gene isolation either (data not shown). Hence a more rigorous bioinformatics approach was employed and a monocot-specific C-terminus probe was generated using existing sequence data for MADS-box putative proteins. The sequence of this probe was the following:

GAGXXVNGXOXXXNXDXXXXOOQXXLKEIAXWXXQNNAOXXDANOLEKLEOLLTOALRNTKXKKMLXOONXG, where X is any amino acid and O is a possible gap. This probe was used to isolate several cereal Type I-like MADS-box sequences from available EST databases and the GenBank non-redundant CDS translated database. Of particular interest were two hits from the barley EST database, chosen based on the presence of a MADS-box domain and the absence of a K-domain, characteristic of Type I MADS-box proteins. These candidates were: BG365393 and TC178280 (TIGR database, http://www.tigr.org) (see Materials and Methods). During the course of our investigations another study characterizing these sequences with respect to their role in the vernalization process was reported [43]. These authors designated these sequences as *ODDSOC1(HvOS1)* and *ODDSOC2(HvOS2),* respectively. In our recent study on the identification and characterization of two *SOC1-like* gene homologues from barley [44] it was shown that two HvSOC1-like proteins containing the K-box domain are closely related to the Type II MADS-box proteins whereas they are more distantly related to ODDSOC1 and ODDSOC2. However, since Greenup et al. (2010) [43] were the first to report these genes we will utilize the nomenclature introduced by these authors and hereafter will be referring to the sequences BG365393 and TC178280 as *HvOS1* and *HvOS2*, respectively.

**Figure 1:**
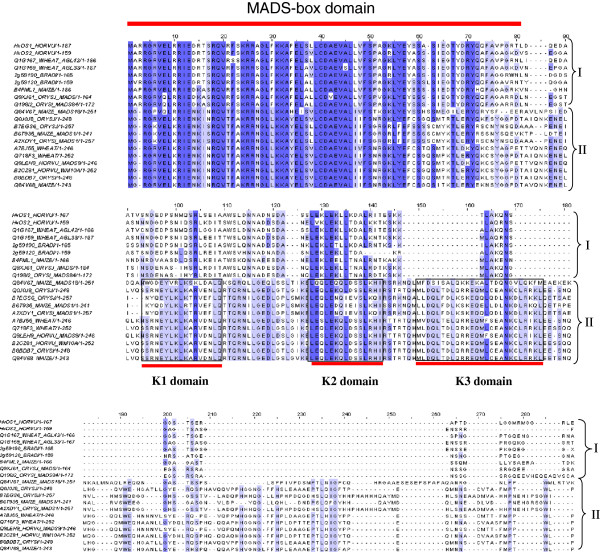
**Amino acid sequence comparisons between Type I MADS-box proteins and Type II MADS-box proteins from different plants.** The name of each sequence consists of its Uniprot ID or GenBank accession number, followed by the species abbreviation according to Uniprot. The K-box domains, K1, K2 and K3 of the Type II MADS-box proteins are shown in boxes and indicated with red bars. The MADS-box domain is also indicated with a red bar. No K-box is present in the Type-I proteins. Identical amino acids are shown in dark blue and similar amino acids in light blue.

**Figure 2:**
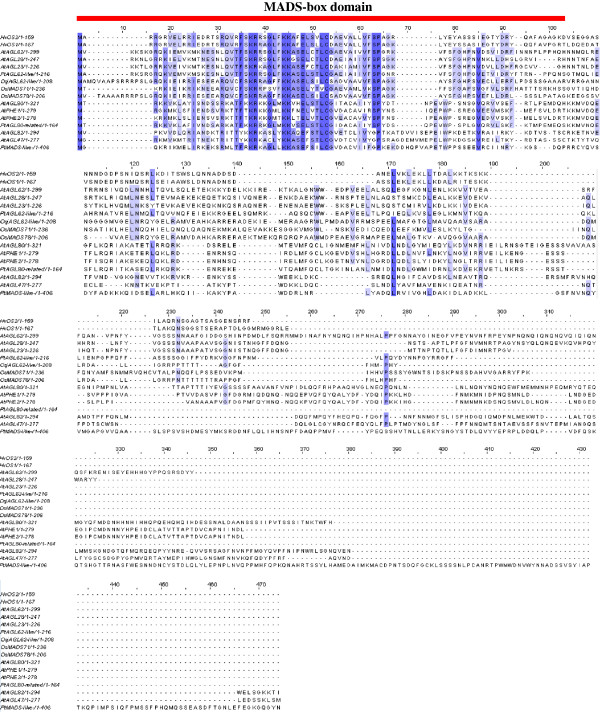
**Amino acid sequence alignments between barley HvOS1, HvOS2, and Type I proteins from different plants.** The name of each sequence consists of its GenBank accession number, also shown in Table 1. Identical amino acids are shown in dark blue and similar amino acids in light blue. The MADS-box domain is indicated with a red bar.

**Figure 3:**
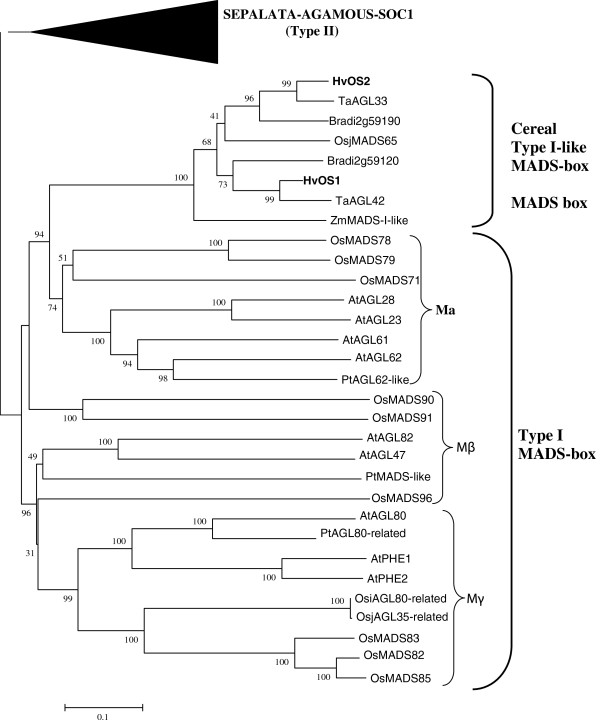
**Phylogenetic tree of MADS-box proteins from different plants.** Phylogenetic tree showing the different clades of MADS-box Type I family proteins. The Type II MADS-box SEPALATA, AGAMOUS and SOC1 families are shown as a condensed branch. The sequences used and their accession numbers are shown in Table 1. Barley HvOS1 and HvOS2 members are in bold. Numbers indicate bootstrap values (1000 = 100%).

*HvOS1* is composed of 834 nt encoding a putative protein of 167 aa, and harboring a 5’UTR of 76 nt and a 3’UTR of 253 nt. *HvOS2* is composed of 1319 nt encoding a putative protein of 159 aa, and containing a 5’UTR of 382 nt, and a 3’UTR of 460 nt. An alignment of MADS-box proteins (Type I and Type II) from different plants is shown in Figure 1. The two barley putative protein sequences HvOS1 and HvOS2 show high similarity to the wheat sequences that have been previously classified as Type I MADS-box sequences [28]. HvOS1 has 96% identity with the wheat TaAGL-42 sequence and HvOS2 shares 92% identity with the wheat TaAGL-33 sequence. HvOS1 and HvOS2 putative protein sequences share 88.9% identity with each other. In Type II MADS-box proteins the characteristic three subdomains of the K-region, K1, K2 and K3 are evident as shown in Figure 1. The barley HvOS1 and HvOS2, wheat TaAGL-33 and TaAGL-42, as well as their closest relatives rice OsMADS65 and maize ZmB4FML1 harbor no regions with similarity to K regions, as expected for Type I MADS-box proteins. A separate alignment of the barley HvOS1 and HvOS2 and different Type I MADS-box sequences was constructed for more clarity (Figure 2). Most of the similarity between the barley HvOS1 and HvOS2 and the other Type I MADS-box sequences is concentrated in the MADS-box domain (first 80 aa), whereas the rest of the sequences show a large degree of divergence. A phylogenetic tree was constructed from a large number of MADS-box proteins from different plants (Figure 3). Three of the subclasses of Type I, Mα, Mβ and Mγ, are shown grouping out into separate clades. The barley HvOS1 and HvOS2 putative proteins, and their putative cereal orthologues, namely, wheat TaAGL-33 and TaAGL-42, brachypodium Bradi2g59190 and Bradi2g59120, rice OsMADS65, and maize ZmB4FML1, cluster together forming a distinct subclass (Figure 3). As mentioned above due to the absence of a typical K-domain which is the hallmark of Type-I proteins, we named these barley sequences as Type I-like HvMADS-box sequences.

### Expression analysis of the Type I-like HvMADS-box genes in different tissues and during seed development

**Figure 4:**
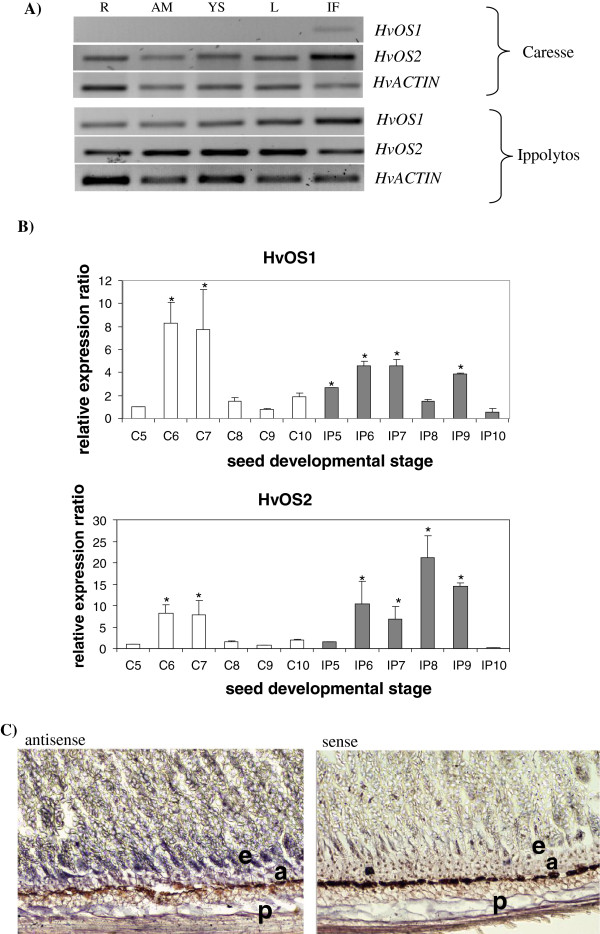
**Expression analysis of barley *Type I-like HvMADS-box *genes. A**) Qualitative RT-PCR expression analysis of *HvOS1* and *HvOS2* barley *Type I-like HvMADS-box* genes. R, roots; AM, apical meristem; YS, young shoots; L, Leaves; IF, immature flower; *HvActin,* was used as the internal control. **B**) Quantitative real-time RT-PCR analysis of *Type I-like HvMADS-box* genes in different seed developmental stages and in two barley cultivars. Expression values were normalized to those of *HvActin*. The relative expression ratio of each sample is compared to the control group which was C5 (Caresse immature flowers). C, cultivar Caresse, (white bars); IP, cultivar Ippolytos (grey bars). 5, Immature flowers; 6, Seed 1–3 DAF; 7, Seed 3–5 DAF; 8, Seed 5–10 DAF; 9, Seed 10–15 DAF; 10, Seed 15–20 DAF. Data represent mean values from two independent experiments with standard deviations. Values significantly different (P < 0.05) from the control group (C5) are marked with an asterisk. **C**) Spatial expression of *HvOS2* in barley seeds. *In situ* localization of *HvOS2* in barley seeds by mRNA *in situ* hybridization analysis. Left, transverse section of developing seeds at 10 DAF hybridized with the antisense probe. Right, transverse section of developing seeds at 10 DAF hybridized with the sense probe, as negative control. a, aleurone; e, endosperm; p, pericarp.

End point PCR analysis showed that *HvOS1* transcripts were detected only in immature flowers in Caresse, whereas they were present in all tissues of Ippolytos examined. Conversely, *HvOS2* transcripts are present in roots, shoots, apical meristem, leaves, and immature flowers in both Caresse and Ippolytos (Figure 4A).

Real time PCR analysis was employed to examine and compare the expression of barley *Type I-like HvMADS-box* genes at different seed developmental stages and in different cultivars, Caresse (a large-seed cultivar) and Ippolytos (a small-seed cultivar) (Figure 4B).

For *HvOS1****,*** an induction of approximately 8 fold in Caresse 1–3 DAF and 3–5 DAF seed, and a decrease thereafter, was observed. In Ippolytos, *HvOS1* was induced in 1–3 and 3–5 DAF seeds by approximately 2 fold. Transcript levels dropped by about 2 fold in 5–10 DAF seeds whereas they increased slightly in 10–15 DAF as compared to immature flowers. In Caresse 15–20 DAF seeds transcript levels increased by about 2 fold, whereas in Ippolytos 15–20 DAF they decreased by about 5 fold (Figure 4B).

A marked increase of approximately 8 fold in *HvOS2* transcript accumulation was observed in Caresse seeds 1–3 DAF and 3–5 DAF, as compared to immature unfertilized flowers. Expression levels dropped thereafter in 5–10, 10–15 and 15–20 DAF seeds to levels comparable to those of unfertilized flowers. In the small-seed cultivar, Ippolytos, *HvOS2* exhibited substantial transcript accumulation in seeds 1–3 DAF and 3–5 DAF. In 5–10 DAF and 10–15 DAF seeds *HvOS2* had a pronounced increase of approximately 10–15 fold, in contrast to Caresse. In Caresse seeds 15–20 DAF there was a slight increase of *HvOS2* transcript. Conversely, a substantial decrease of about 10 fold was seen in Ippolytos 15–20 DAF seeds (Figure 4B). Expression of *HvOS2* was examined in another large-seed cultivar, Byzantio. Similar to Caresse, *HvOS2* transcript levels were significantly lower in 5–10, 10–15 DAF seeds as compared to Ippolytos (Additional File 2).

More detailed expression of the *HvOS2* gene within the seed was examined by *in situ* localizations using cross sections of seeds and DIG-labelled antisense probe. Strong hybridization signal was observed in the aleurone layer and the first layer of endosperm cells adjacent to the aleurone layer that was absent from the sense-control (Figure 4C), suggesting cell-specific expression in the endosperm. In addition, strong hybridization signal was observed in embryo cells between sections hybridized with antisense as well as sense probes suggesting no specific expression in the embryo. *HvOS1* analysis was not possible due to lack of appropriate sequences specific for the *HvOS1* gene satisfying the particular needs of this experiment.

### Expression analysis of the Type I-like HvMADS-box genes in response to JA and ABA

**Figure 5:**
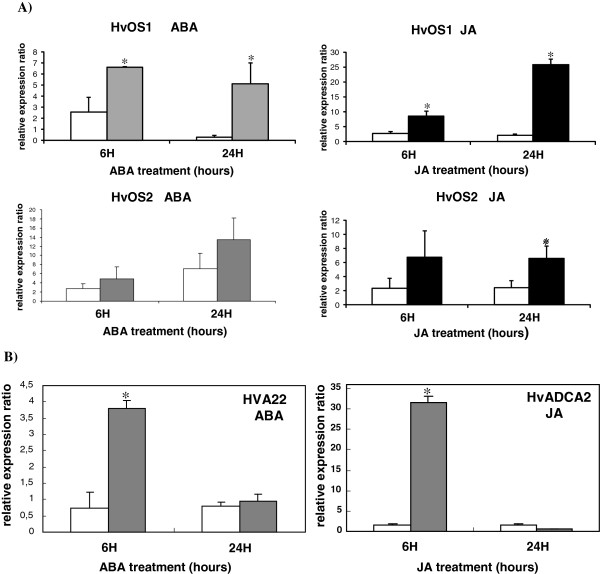
**Expression analysis of *HvOS1, HvOS2 *after treatment of seedlings with ABA and JA. A**). Quantitative real-time PCR analysis of *HvOS1* and *HvOS2* genes at 6 and 24 h after treatment of Caresse seedlings with 100 μM ABA and JA respectively. Grey and black bars, ABA and JA hormone-treated plants, respectively; white bars, no hormone treated plants (mocked with H_2_O/Tween for 6 h and 24 h, respectively). **B**) Quantitative real-time PCR analysis of the barley genes *HVA22* (known to be induced by ABA) and *HvADC2* (known to be induced by JA) at 6 and 24 h after treatment of Caresse seedlings with 100 μM ABA and 100 μM JA, respectively, used as positive controls. Expression values were normalized to those of *HvActin.* Data represent mean values from two independent experiments with standard deviations. Relative expression ratio of each sample was compared to the control group which was untreated plants, 0 h, and was assigned the value of 1. Values significantly different (P < 0.05) from the control group (untreated 0 h) are marked with an asterisk.

Since JA and ABA responsive elements were detected on the promoters of both genes (see below, Figure 7) quantitative real time PCR was performed using total RNA from seven-day-old seedlings subjected to exogenous application with JA and ABA in order to examine whether expression of the *Type I-like HvMADS-box* genes responds to these hormones. *HvOS1* showed a significant increase of approximately 2.5 fold and 10 fold at 6 h and 24 h, respectively, after application of ABA, as compared to the control samples. *HvOS2* did not display statistically significant expression changes after ABA treatments (Figure 5A).

**Figure 6:**
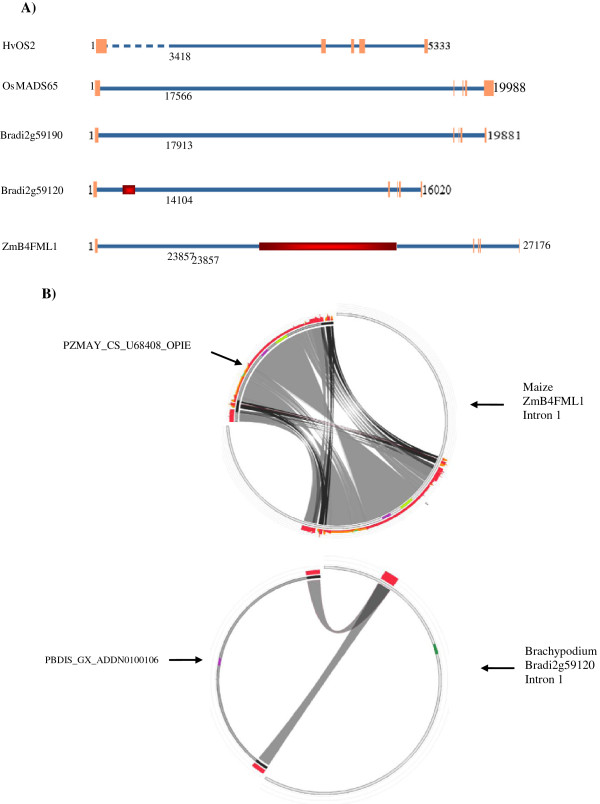
**Genomic organization of *HvOS2 *in relation to its cereal homologues. A**) Schematic view of gene bodies (exons plus introns) of five cereal *MADS-I-like genes*. Exons are depicted with orange boxes and introns with blue lines. Numbers on start and end of contigs refer to the length of particular genomic region. Length (bp) of first large introns is given under each intron. Regions within the first intron where retrotransposons were found are highlighted in red. **B**) Sequence similarity of the first introns of maize *ZmB4FML1* and brachypodium *Bradi2g59120* with retrotransposons, visualized in Circoletto (http://tools.bat.ina.certh.gr/circoletto/) after blast analysis with MASiVE (http://tools.bat.ina.certh.gr/masive/) and LTRphyler (http://tools.bat.ina.certh.gr/ltrphyler/). Upper circle: Left, full length maize retrotransposon *Copia. PZMAY_CS_U68408_OPIE* detected within the first intron of maize *ZmB4FML1* and depicted in red; Right, the first intron of maize *ZmB4FML1*. The region of retrotransposon homology is shown in red. Bottom circle: Left, the brachypodium retrotransposon, *Gypsy PBDIS_GX_ADDN0100106*. Right, the first intron of brachypodium *Bradi2g59120*. Regions of homology are shown in red. Coding regions of retrotransposons integrase and reverse transcriptase are in purple and yellow-green, respectively. The 5’and 3’ LTR regions of retrotransposons are shown with bold black lines. Dark grey and light grey interconnecting ribbons depict regions of high and low similarity, respectively.

*HvOS1* showed a significant induction of about 3 fold and 12 fold at 6 h and 24 h, respectively, after JA treatment, as compared to the control. The *HvOS2* transcript was increased by about 3 fold at 24 h after JA treatment (Figure 5A). Two barley genes *HVA22* and *HvADC2* known to be induced by ABA and JA, respectively, were used as positive control genes for the ABA and JA treatments (Figure 5B).

### Genomic organization

Analysis of the genomic structure of the cereal *Type I-like MADS-box* homologues was performed by retrieving the genomic sequences of *OsMADS65*, *Bradi2g59190, Bradi2g59120,* and *ZmB4FML1* via BLAST in the Phytozome database (http://www.phytozome.net/) using the full length ESTs. Partial *HvOS2* genomic sequence was identified on two different contigs after a search in the barley database http://webblast.ipk-gatersleben.de/barley/index.php. No genomic data are available, thus far, for *HvOS1.* The organization of exons and introns of all five genes was then identified using the Spidey tool in NCBI (http://www.ncbi.nlm.nih.gov/spidey). The genomic organization of the cereal *Type I-like MADS-box* homologues was found to be very similar, all five genes containing five exons interrupted by four introns of approximately the same size and relative positions (Figure 6A and Additional File 3). A remarkably large intron of 17566 bp, 17913 bp, 14104 bp and 23857 bp, for *OsMADS65*, *Bradi2g59190*, *Bradi2g59120* and *ZmB4FML1*, respectively, interrupts exon 1 and exon 2 in all four genes. The two contigs carrying *HvOS2* are not overlapping thus the first intron was not obtained fully (Figure 6A). Since *HvOS2* exon 1 and the beginning of intron 1 reside on non-overlapping contigs, the 5333 bp represent the minimum length of the genomic fragment harboring all exons and introns and the 3418 bp the minimum length of the first intron. The large size of the first introns intrigued us into inspecting further these sequences for the presence of retroelements. Recently our group has developed advanced *in silico* methods (http://tools.bat.ina.certh.gr/masive/) (http://tools.bat.ina.certh.gr/ltrharvester/) for detecting and analyzing retrotransposons [45][46]. Using these methods a 8993 bp region (10946–19938 bp) in the middle of the *ZmB4FML1* first intron was found to have high similarity with the full length of the *Copia PZMAY_CS_U68408_OPIE* retrotransposon, which is a Sirevirus element, (Figure 6B) and especially at the long terminal repeats (LTR). The 3’LTR corresponds to a 1277 bp region (10946–12222 bp) and the 5’LTR spans a 1253 bp region (18686–19938 bp) within the *ZmB4FML1* first intron sequence. Similarly, a 543 bp region (1972–2515 bp) in the beginning of the first intron of *Bradi2g59120* displayed high similarity with the *Gypsy PBDIS_GX_ADDN0100106* retrotransposon, at regions encompassing the 5’LTR (1–529 bp) and 3’LTR (10500–11044 bp), respectively (Figure 6B). Homology to retrotransposons was not observed for any other genomic region of *ZmB4FML1* and *Bradi2g59120*. No homology with retrotransposons was found in the *Bradi2g59190*, *OsMADS65* and *HvOS2* genes.

**Figure 7:**
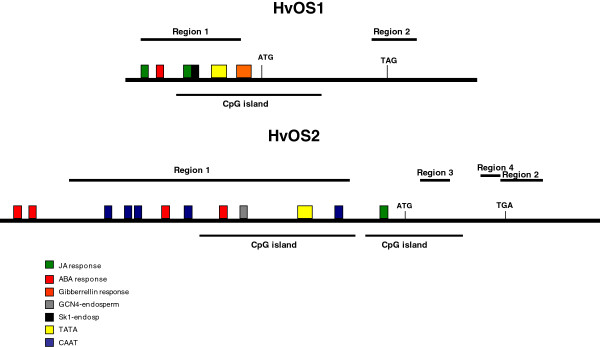
**Schematic diagram of 5’ upstream and 3’ downstream regions of *HvOS1 *and *HvOS2. *** Regions 5’ upstream from the ATG translation initiation site, coding regions, and 3’downstream regions are depicted with thick bold black lines. Translation initiation codons, ATG, and stop codons TAG and TGA, respectively, are indicated. *Cis* acting elements are depicted in colour-coded boxes. Predicted CpG islands and regions used in DNA methylation assays are also depicted with thin black lines.

Greenup et al. (2010) have assigned a position for *HvOS1* and *HvOS2* on barley chromosome 3 H, in syntenic regions to Brachypodium and rice, and proximal to the unigene 17348 [43]. Analysis in HarvEST found unigene 17348 to reside close to the POPA3_0092 SNP marker on the comparative barley maps. This position is in proximity to important QTL markers for seed quality traits such as grain protein content (QGpc.StMo-3 H) and grain yield (QYld.StMo-3 H) (Additional File 4).

### Presence of regulatory elements in the 5’ upstream regions of Type I-like HvMADS-box genes

5’ upstream sequences of the *Type I-likeHvMADS-box* genes were isolated (542 bp 5’ from the ATG site for *HvOS1,* and 2185 bp 5’ from the ATG site for *HvOS2*) using genome walking in the cultivar Caresse. 5’ upstream regions of these genes were interrogated for known *cis*-acting regulatory elements and results were analyzed with special attention to elements identified and characterized in cereals. The 5’ upstream region of both *HvOS1* and *HvOS2* genes showed the presence of endosperm-specific-elements, and ABA and JA responsive elements (Figure 7 and Additional File 5). *HvOS1* also contains a gibberellin-responsive-element. TATA and CAAT boxes were also detected. Further inspection of these sequences was carried out in order to identify regions which may be prone to cytosine-methylation. Predicted CpG islands which may play important roles in the regulation of gene expression via DNA methylation-directed silencing were identified in both sequences (Figure 7 and Additional File 5).

### DNA methylation differences in the 5’ and 3’ flanking regions of Type I-like HvMADS-box genes

In order to examine the DNA methylation pattern of the *Type I-likeHvMADS-box* regulatory regions and uncover potential links to gene expression differences among different developmental stages, we performed *Mcr*BC digestions of genomic DNA and subsequent PCR amplification analysis. *Mcr*BC is a methylation-dependent restriction enzyme which digests DNA that is methylated at two or more cytosines, thus reducing PCR amplification of a selected fragment proportionally to the methylated cytosines [47]. Therefore the presence or absence (as well as reduction) of amplicons after PCR amplification of *Mcr*BC-digested genomic DNA with primers specific for a particular region suggests lower or higher degree of methylation within this region, respectively. We chose two developmental stages with the largest differences in expression, Caresse immature flowers and 1–3 DAF seeds.

**Figure 8:**
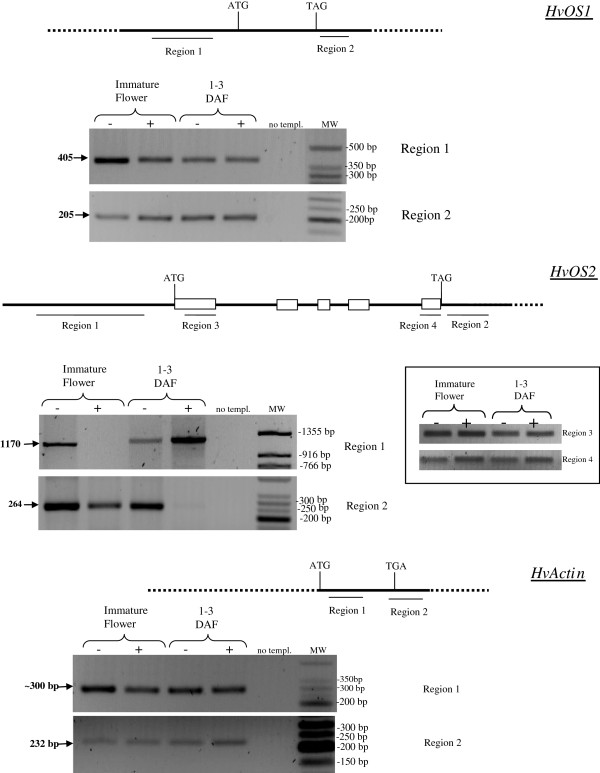
**DNA methylation assays in seed stages. ** Genomic DNA was digested with *Mcr *BC and PCR-amplification followed. (−), no *Mcr*BC; (+), digestion with *Mcr*BC; IF, immature flower; 1–3 DAF, 1–3 days after fertilization. The regions analyzed are depicted schematically above the gel images for each gene. White boxes represent exons.

In *HvOS1* there was less degree of amplification of the 5’upstream region (region 1) in *Mcr*BC-digested than undigested genomic DNA in immature flower, whereas there was no difference in 1–3 DAF seeds (Figure 8, upper panel), implying more methylation sites in immature flowers than 1–3 DAF seeds. This might account for reduced expression of the gene in immature flowers. Amplification of the 3’ downstream region (region 2) in *Mcr*BC-digested DNA was not reduced compared to undigested genomic DNA either in immature flowers or in 1–3 DAF seeds implying low methylation in this region (Figure 8, upper panel).

For *HvOS2,* two non-coding regions, a 1170 bp region [located at −1525 bp upstream from the ATG site (region 1)], and a 264 bp region located 3’downstream from the coding region (region 2), and two coding regions (region 3 and region 4) were analyzed (Figure 8, middle panel). PCR amplification of immature flower genomic DNA digested with *Mcr*BC did not produce the expected 1170 bp upstream fragment contrary to the digested sample, suggesting the presence of methylated sites within region 1 in immature flower genomic DNA. The 1170 bp amplicon was produced from both *Mcr*BC-digested and undigested genomic DNA from 1–3 DAF seeds suggesting a lower degree of methylation within region 1 in 1–3 DAF genomic DNA. The presence of higher DNA methylation level in the promoter of *HvOS2* in immature flowers may be associated with downregulation of this gene in this tissue as compared to the high levels of expression in 1–3 DAF seeds. Examination of the 3’ downstream region (region 2) revealed that the expected fragment was amplified after *Mcr*BC-PCR amplification of immature flower genomic DNA although to a lesser extent than the undigested sample (Figure 8, middle panel). Interestingly, amplification of *Mcr*BC-digested 1–3 DAF genomic DNA produced nearly no amplicon, implying a much higher degree of DNA methylation in the 3’downstream region of *HvOS2* in 1–3 DAF than in immature flowers. Finally, examination of two sites within the coding region, in Exon 1 and Exon 5 (region 3 and region 4), did not show any decrease of PCR amplification in *Mcr*BC-digested as compared to undigested DNA, either in immature flowers or in 1–3 DAF (Figure 8, middle panel-inlet).

Two regions were also examined within the *HvActin* gene, one within the coding region (region 1) and one in the 3’region downstream from the coding region (region 2). There were no obvious differences in the amplification of these regions between *Mcr*BC-digested and non-digested genomic DNA, suggesting no methylation sites in these fragments (Figure 8, bottom panel). The larger than expected size of the PCR product after amplification of region 1 (300 bp instead of 235 bp) implies the presence of a small intron in that region.

**Figure 9:**
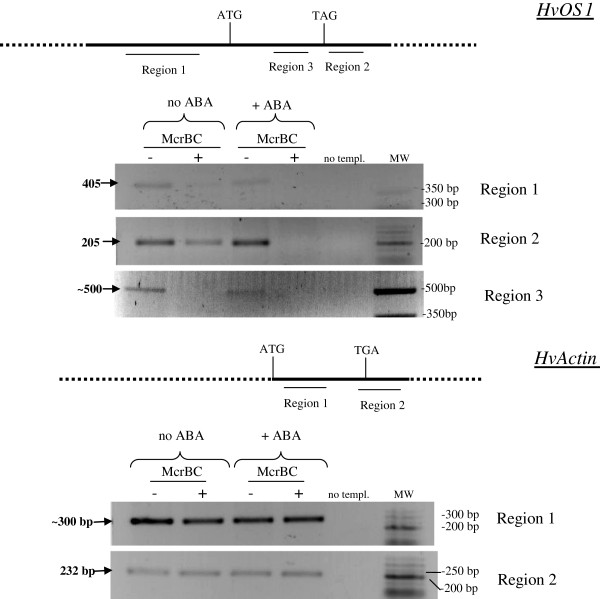
**DNA methylation assays after ABA treatment.** Genomic DNA from untreated and ABA-treated seedlings, at 24 h after treatment, was digested with *Mcr*BC and PCR-amplification followed. (−), no *Mcr*BC; (+), digestion with *Mcr*BC; The regions analyzed are depicted schematically above the gel images.

Genomic DNA methylation assays were also performed for *HvOS1* in ABA-treated and untreated seedlings (Figure 9). *HvOS1* is induced upon ABA expression (Figure 5A). Interestingly, examination of a *HvOS1* 3’UTR region (region 2) revealed no PCR amplification in the *Mcr*BC-digested genomic DNA from 24 h ABA-treated seedlings, suggesting the presence of methylation sites in this region. Less PCR amplification was observed in *Mcr*BC-digested than undigested genomic DNA in untreated seedlings suggesting a lower degree of methylation in untreated than ABA-treated seedlings (Figure 9). In region 1 of the promoter, nearly no amplification is detected in ABA-treated and non ABA treated *Mcr*BC-digested DNA, suggesting DNA methylation in this region. A third fragment within the coding region (region 3) was also tested and showed no PCR amplification in *Mcr*BC-digested DNA from both ABA-treated and non ABA-treated seedlings implying DNA methylation in this region with and without ABA. The larger than expected size of the PCR product after amplification of region 3 (500 bp instead of 184 bp) implies the presence of a small intron in that region.

## Discussion

In the current study we present the identification, protein sequence structural analysis, phylogenetic characterization, expression profiles, genomic organization and promoter analysis of two genes encoding Type I-like MADS-box proteins in barley. During the course of our studies Greenup et al. (2010) reported the identification of these genes in barley and named them *HvOS1* and *HvOS2*[43]. These authors focused their investigations on the role of these genes in the vernalization response and proposed a cereal-specific pathway controlling vernalization-induced flowering in temperate cereals different from the one reported for Arabidopsis.

We have focused our study on detailed *in silico* structural analysis of HvOS1 and HvOS2 protein sequences and on gene expression during seed development, in different cultivars, and in response to the seed developmental- and stress-related hormones ABA and JA. Moreover we have examined 5’ and 3’ regulatory regions of *HvOS1* and *HvOS2* for differential DNA methylation patterns that may be associated with differential gene expression.

### Protein sequence analysis

Both *HvOS1* and *HvOS2* encode proteins possessing a MADS-box domain, lacking the typical K-box of Type II MADS-box proteins, and having highest resemblance to two Type I MADS-box proteins from wheat, TaAGL-33 and TaAGL-42, respectively. Phylogenetic analysis showed that the two barley Type I-like MADS-box proteins together with their putative orthologues from wheat (TaAGL-33 and TaAGL-42), brachypodium (Bradi2g59190, Bradi2g59120), rice (OsMADS65) and maize (ZmB4FML1), form a cluster which is closer related to the alpha clade of Type I MADS-box proteins (Mα). Interestingly this group does not contain sequences from Arabidopsis or other dicots.

### Differential expression during vegetative, reproductive and seed development

Qualitative RT-PCR expression analysis in different tissues showed that the *HvOS1* transcript was expressed in roots, apical meristem, young shoots, leaves, and immature flowers in Ippolytos but was present only in immature flowers in Caresse and was not detected in the other tissues examined. On the other hand, the *HvOS2* transcript is present in roots, apical meristem, young shoots, leaves of seedlings and in immature flowers in two different barley cultivars, Caresse and Ippolytos. In that sense, *HvOS1* expression in Ippolytos and *HvOS2* expression in both cultivars resembles the Arabidopsis *Type I MADS-box* gene *AGL28* gene which is expressed widely in vegetative and reproductive tissues [18].

*Type I MADS-box* genes have been shown to be expressed in the female gametophyte and the developing seeds in Arabidopsis and rice and have been assigned crucial roles in seed development through functional characterization in Arabidopsis. In order to start understanding the role of the two barley *Type I-like MADS-box* genes in seed development we conducted quantitative real-time PCR analysis to examine their expression in unfertilized flowers and during different seed developmental stages in two barley cultivars with varying seed size, Caresse (large seed) and Ippolytos (small seed). Quantitative real time PCR analysis demonstrated significant differences in gene expression among different seed developmental stages within a cultivar and between two cultivars with different seed size. In particular, both genes exhibited a remarkable induction post-fertilization, in 1–3 DAF and 3–5 DAF seeds in Caresse (approximately 8 fold) and a decline at the later stages of seed development. Likewise, an increase in transcript levels was evidenced for both genes in 1–3 DAF and 3–5 DAF seeds, in Ippolytos. However, in Ippolytos a remarkable increase was also observed for the *HvOS2* gene at the later seed stages of 10–15 DAF and 15–10 DAF and a large reduction in 15–20 DAF, whereas such changes were not observed in Caresse.

The expression pattern of *Type I-like HvMADS-box* genes agrees with the expression of most *Type I MADS-box* genes in Arabidopsis, where they were found to be induced in early syncytial endosperm up to the 4 DAF seed developmental stage [8,18,48]. *HvOS2* resembles its closest wheat homologue, *TaAGL33*, being expressed in 6–19 DAF seeds as well as in roots and leaves of seedlings [28]. On the other hand, expression of the wheat *TaAGL42*, the closest homologue to *HvOS1*, is not detected either in immature flowers or in 6–19 DAF seeds of wheat, implying a different role for this homologue in wheat at least in the particular cultivar studied. The rice *OsMADS65* homologue has been reported to be widely expressed [9]. Microarray and quantitative PCR analysis has generated an expression profile for the *Type I MADS-box* genes in rice [9]. Most genes are expressed, during the vegetative and reproductive stages and during seed development. Interestingly, quantitative PCR analysis showed that three genes of the Mα clade, *OsMADS71*, *OsMADS78* and *OsMADS79*, have a substantial induction in 5–10 DAF and 10–15 DAF seeds resembling the expression pattern of *HvOS2* in the Ippolytos cultivar. *OsMADS71*, *OsMADS78* and *OsMADS79* are the closest rice homologues to the barley *Type I-like HvMADS-box* genes, after *OsMADS65*. Protein sequence similarity together with similar expression patterns may reflect similar functional roles for these homologues in these two closely related cereal species.

Significant differences in gene expression were observed between Caresse, a large-seed cultivar and Ippolytos, a small-seed cultivar. A striking increase in *HvOS2* transcript was found in Ippolytos 5–10 DAF seeds (~20fold) and 10–15 DAF seeds (~15fold) as compared to the same seed developmental stages in Caresse, suggesting cultivar-dependent differential gene expression. An increase in *HvOS1* transcript was also evidenced in Ippolytos 10–15 DAF seeds (~4fold) as compared to the same seed stage in Caresse. Similar to Caresse, examination of *HvOS2* expression in the large-seed cultivar, Byzantio, also found significant differences (10–15 fold) at the 5–10 and 10–15 DAF seed stages between Byzantio and the small-seed cultivar Ippolytos, supporting a possible association of *HvOS2* expression and seed size.

Differential expression of *HvOS1* and *HvOS2* (pronounced at the 5–10 and 10–15 DAF seed stages) between Caresse (a large-seed cultivar) and Ippolytos (a small-seed cultivar) may suggest an association of these genes with the size of seed. In barley, endosperm cellularization begins at approximately 4 DAF and ends at 6–8 DAF, when the seed maturation process begins [1,2,49]. It may be possible that the differences in *Type I-like HvMADS-box gene* expression between the two cultivars during these critical stages of endosperm development are associated with the processes of cellularization and seed filling and ultimately with grain size. Certainly, a large number of genotypes with different seed size should be further tested to conclusively assess any association of these genes with the size of seed.

*In situ* hybridization experiments localized *HvOS2* in the aleurone layer and in endosperm-specific cells situated adjacent to the aleurone layer, in 10 DAF seeds. Specific expression of this gene within the endosperm may be associated with particular gene expression programmes involved in seed maturation.

### Genomic organization

Analysis of the genomic organization of the *HvOS2* revealed resemblance with its closest cereal homologues *OsMADS65*, *Bradi2g59190, Bradi2g59120* and *ZmB4FML1*. Extensive conservation in the exon-intron organization is evident among all five genes with five exons and four introns at approximately the same relative positions and approximately the same size except from the fifth exon of the maize gene *ZmB4FML1*. Genomic organization for *HvOS1* was not possible due to lack of barley sequence information. With the rapid advancement in the sequencing of the barley genome [50,51] the complete genomic sequence of both *HvOS1* and *HvOS2* will become available soon for further analysis.

A strikingly large first intron is present in all five cereal homologues studied. In *OsMADS65*, *Bradi2g59190, Bradi2g59120, ZmB4FML1* this intron accounts for about 85-90% of the entire length of the gene bodies (here exons plus introns). Notably, our analysis showed that the maize *ZmB4FML1* intron has high similarity to a full length maize Sirevirus retrotransposon, *Copia PZMAY_CS_U68408_OPIE.* Likewise, a fragment of the first intron of the *Bradi2g59120* gene exhibits high similarity to remnants of *PBDIS_GX_ADDN010010,* a brachypodium *Gypsy* retrotransposon. Homology with retrotransposons was not detected in the first intron of barley *HvOS2*, thus far.

Recently a sensitive comparative analysis of the genomes of the plant-specific Sireviruses retrotransposons was performed [45,46], in an effort to understand their significance in the evolution of their plant hosts and their putative role in the epigenetic regulation of plant genes. In addition, our group reported a systematic analysis of >10,600 intact and ~28,000 degenerate maize Sireviruses [52] discovered by the MASiVE algorithm in maize, and showed that Sireviruses account for 90% of the Copia type population, comprising 21% of the maize genome, and reside in near-gene areas. The element *PZMAY_CS_U68408_OPIE* found within the intron of the maize *ZmB4FML1* gene belongs to *Opie,* one of the most abundant families of the *Copia* genera and its location within an intron may suggest additional insertion sites of Sireviruses within genes.

It has become more and more evident that intact or remnants of transposable elements may be implicated in the transcriptional regulation of flowering plants especially in the control of gene imprinting [5,53]. Most imprinted genes, mainly expressed only from the maternal allele, are found in the female gametophyte and in the endosperm of the developing seed [54]. Massive demethylation of transposable elements and regions generating siRNAs was shown to take place in the Arabidopsis endosperm as opposed to the embryo [53,55] and it has been hypothesized that fragments of transposable element inserted near genic regulatory elements may guide DNA methylation at specific alleles and govern imprinted gene expression (for example silencing of paternal alleles) [4,56]. Presence of transposable elements within introns in genes of cereals may perform similar regulatory functions.

*In silico* mapping of *HvOS1* and *HvOS2* identified a corresponding SNP marker on barley chromosome 3 H close to regions associated with important agronomical traits such as grain protein content and grain yield. This could prove important for the development of functional markers to be used in marker assisted selection for breeding programmes.

### Promoter elements

Promoter sequence analysis revealed putative CpG regions within the 5’upstream regions of both *HvOS1* and *HvOS2*. In addition several regulatory elements were identified, including elements related to endosperm and seed development such as endosperm-specific elements and ABA and gibberrellin-responsive elements. These observations together with the expression patterns at different seed developmental stages and in response to the seed development-related hormone ABA, mentioned above, reinforces the suggestion for functional roles for these genes in barley seed development.

JA responsive elements were also identified on the promoters of both *HvOS1* and *HvOS2*. ABA and JA elements identified in the 5’upstream regions of *HvOS1* and *HvOS2* were also detected in the promoters of several of the rice homologues such as *OsMADS65* and *OsMADS84* and brachypodium homologues, *Bradi2g59190* and *Bradi2g59120* (data not shown)*.* The hormones JA and ABA are known to mediate abiotic stress responses in plants. It is possible that *Type I-like HvMADS-*box genes are responsive to ABA- or JA- associated stress conditions. In support to this the *TaAGL42* homologue is induced by cold stress in two winter wheat cultivars [57] and a number of *Type I-MADS-box* genes in rice were found to be induced by abiotic stress such as cold, drought, and increased salinity [9].

### DNA methylation

DNA methylation analysis was undertaken to reveal whether differentially methylated regions exist in the 5’upstream and 3’downstream regions of *HvOS1* and *HvOS2* between two different seed developmental stages with different gene expression levels. In particular, using the *Mcr*BC-PCR assay it was shown that the 5’upstream region of *HvOS2* is highly methylated in Caresse immature flowers as compared to 1–3 DAF seeds where lower degree of methylation was observed. It is likely that the observed downregulation of *HvOS2* in immature flowers may be due, in part, to a DNA methylation silencing effect. Similarly, a higher degree of DNA methylation in the 5’ upstream region of *HvOS1* in immature flowers as opposed to low DNA methylation in 1–3 DAF seeds suggests that downregulation of *HvOS1* in immature flower maybe be partially due to a DNA methylation repressive mechanism. Notably, a genome-wide analysis in Arabidopsis demonstrated extensive global DNA demethylation in 5’ upstream and 3’ downstream regions in endosperm but not in embryo tissue and this is associated with gene activation in endosperm and gene silencing in the embryo, respectively [55]. A similar epigenetic mechanism could be operating in cereals seeds. The endosperm in monocot species (like cereals) is not consumed by the embryo during seed development as is the case in dicots, but rather develops to the mature seed which contains storage proteins and starch and constitutes the nutritional part of the seed. Although our study did not separate embryo from endosperm tissue, we may assume that the seed of 1–3 DAF in barley is mainly represented by endosperm syncytium with very small contribution from the embryo. Further studies will enable us to draw any parallels between dicots and monocots concerning DNA methylation and endosperm development.

DNA methylation in the 3’downstream region of *HvOS2* was found to be increased in 1–3 DAF seeds where the gene is highly induced, than in immature flowers where the gene is downregulated. This resembles the methylation status of the Arabidopsis *PHE1* where the 3’downstream region of the expressed paternal allele was found to be methylated, whereas the silenced maternal allele was unmethylated in the same region [22].

Since *HvOS1* was found to be induced upon ABA treatment we investigated whether DNA differential methylation is evidenced in ABA treated and untreated seedlings. Notably, examination of the *HvOS1* 3’UTR region (region 2) in ABA-treated seedlings revealed higher degree of DNA methylation in leaves of ABA-treated seedlings than in untreated seedlings. This is the first time that DNA methylation has been investigated in MADS-box genes in association to hormonal treatment. It is likely that differential DNA methylation may be associated to differential expression of this barley gene in response to ABA.

Chromatin Immunoprecipitation (ChIP) experiments performed by Greenup et al. (2010) demonstrated the presence of the H3K4me3 gene activation mark, deposited by a Trithorax (TrX) methyltransferase, and found higher H3K4me3 in non vernalized than vernalized tissue, in agreement with *HvOS2* decreased expression upon vernalization [43]. They also demonstrated the presence of H3K27me3 mark (the repressive histone modification mark deposited by the PRC2 complex) on the 5’ UTR region of *HvOS2,* though levels were low both in vernalized and non vernalized tissue. This may be related to the fact that DNA methylation may exclude sites from H3K27me as mentioned in Weinhofer et al. 2010 [58]*.* The observations by these authors together with our current results suggest that *HvOS1* and *HvOS2* are targets of epigenetic regulation by histone modifications or DNA methylation.

Epigenetic regulation of endosperm-related genes such as *Type I MADS-box* genes and *PRC2* Polycomb group genes like *MEA*, through DNA methylation and/or histone modifications, has been well documented in Arabidopsis especially for *PHE1*, *AGL62* and *AGL36* and MEA [22,26,27,59]. Our current results on barley *Type I-like MADS-box* genes point to a role for these genes in endosperm development. Moreover, differences in DNA methylation at different seed developmental stages with different gene expression suggest epigenetic regulation of endosperm-associated genes in barley. In support to this, our group has recently characterized barley epigenetic regulators such as histone deacetylases (*HDACs),* histone acetyltransferases (*HATs), PRC2* Polycomb group genes*, Trithorax (TrX)* histone methyltransferase and histone demethylase *(HDM)* genes*,* and investigated their roles during seed development [30,44,60-64].

## Conclusions

In the present work two *Type I-like MADS-box* gene homologues were studied during seed development in barley. *HvOS1* and *HvOS2* are related to *Type I MADS-box* genes and exhibit differential expression in particular seed developmental stages in a cultivar-specific manner. *HvOS1* is induced by the phytohormone ABA and *HvOS2* is detected in specific endosperm sub- compartments. Together these observations point to a role for *HvOS1* and *HvOS2* in seed development. DNA methylation differences associated with differences in gene expression are suggestive of epigenetic regulation of *HvOS1* and *HvOS2* in barley, in accordance to Arabidopsis *Type I MADS* genes. The current study provides further important knowledge for the understanding of gene regulation during seed development in barley and other agronomically important cereals.

## Authors’ contributions

AK conceived and designed the experiments, performed the qualitative and quantitative real time PCR assays, the DNA methylation experiments and promoter analysis and wrote the manuscript. CE performed tissue sampling and genome walking experiments. VD performed tissue sampling, and DNA methylation experiments. CK performed most of the *in situ* hybridization experiments. ET performed *in situ* hybridization experiments and participated in revising the manuscript. AT performed protein alignments, phylogenetic analysis, and genomic organization analysis. EK participated in the *in situ* analysis. IG participated in the bioinformatics analysis and *in silico* mapping. EF designed and supervised the *in situ* localization experiments. AST conceived and directed the whole study, and participated in the writing and revising of the manuscript. All authors read and approved the final manuscript.

## Supplementary Material

Additional file 1 Table S2.Primers used in expression and DNA methylation analysis. Click here for file

Additional file 2 **Quantitative real time PCR expression analysis of *HvOS2 *in the large-seed cultivars, Byzantio, and a small-seed cultivar, Ippolytos, at different stages of seed development.** Click here for file

Additional file 3 **Detailed exon/intron organization of the *HvOS2 *, *Bradi2g59190, Bradi2g59120, OsMADS65, *and *ZmB4FML1. *** Click here for file

Additional file 4 **Mapping *in silico. *** Click here for file

Additional file 5 **Analysis of 5’ upstream regions of *HvOS1 *and *HvOS2. *** Click here for file
