# Supplementary material for: The study of two barley Type I-like MADS-box genes as potential targets of epigenetic regulation during seed development
Source: BMC Plant Biol. 2012 Sep 17;12:166. doi: 10.1186/1471-2229-12-166 (PMC3499179; doi:10.1186/1471-2229-12-166)
Supplement: Additional file 1 — Table S2.Primers used in expression and DNA methylation analysis. [file 1471-2229-12-166-S1.doc]

**Additional File 1:**

**Table 2. Primers used for expression analysis and DNA methylation assays**

| **Primer name** | **5’ - 3’ Primer sequence** | **Size of fragment (bp)** |
| --- | --- | --- |
| **Expression analysis** |  |  |
| *HvOS1* F1 | AAGGACCCTGGATCAAGAAG | 184 |
| *HvOS1* R1 | CTTCTTAGATTCTGTAATTCTCAG |  |
| *HvOS2* F1 | AAGGTTTTGAGCAGCCATG | 264 |
| *HvOS2* R1 | CTGATATGTTAATATGAGCATCACAC |  |
| HvActin F | CTGACGGTGAGGACATCCAG | 235 |
| HvActin R | CGTGAGGATACCTCTCTTGGA |  |
| HVA22 F | TGGCGCTCCCGCAGTTCAAG | 123 |
| HVA22 R | CCTTGAGTATGTGCACCTTGTG |  |
| HvADC2 F | CTTCCACATCGGCTCCATGATC | 237 |
| HvADC2 R | TTGAGCCGCACCGCCTGCAC |  |
| *HvOS2 in situ* F | AGAAGGTTTTGAGCAGCCATG | 202 |
| *HvOS2 in situ* R | *CCAAGCTTC***ATTAACCCTCACTAAAGGGAGA**AACAAGGACAC |  |

| **DNA methylation assays** |  |  |
| --- | --- | --- |
| *HvOS1* F1 m (5’) | TTATTCGGATGCCAATGAGC | 405 |
| *HvOS1* R1 m (5’) | TGGATCGAAGGGGTAACAAAA |  |
| *HvOS1* F2 m (3’) | GAATGGGAGGAAGGCTTGAG | 205 |
| *HvOS1* R2 m (3’) | GACCCGATGCATCAGTATTAAC |  |
| *HvOS1* F3 m (coding) | AAGGACCCTGGATCAAGAAG | 184 |
| *HvOS1* R3 m (coding) | CTTCTTAGATTCTGTAATTCTCAG |  |
| *HvOS2* F1 m (5’) | AAGAGTGCAGAGGAAGGGGAG | 1170 |
| *HvOS2* R1 m (5’) | CGAGCCCATTGTTTTGCTTA |  |
| *HvOS2* F2 m (3’) | AAGGTTTTGAGCAGCCATG | 264 |
| *HvOS2* R2 m (3’) | CTGATATGTTAATATGAGCATCACAC |  |
| *HvOS2* F3 (exon 1) | GATCGAGGACCGGACGAG | 152 |
| *HvOS2* R3 | TGGAGGAGGCGTACTCGTAG |  |
| *HvOS2* F4 (exon 5) | AGATATTGGCGCAACGAAAT | 78 |
| *HvOS2* R4 | ACATGGCTGCTCAAAACCTT |  |
| HvActin F1 m | CTGACGGTGAGGACATCCAG | 235 |
| HvActin R1 m | CGTGAGGATACCTCTCTTGGA |  |
| HvActin F2 m | CGATCGTCCACAGGAAGTGC | 232 |
| HvActin R2 m | CACAAACACACTGACAATGCAC |  |
