# Supplementary material for: The study of two barley Type I-like MADS-box genes as potential targets of epigenetic regulation during seed development
Source: BMC Plant Biol. 2012 Sep 17;12:166. doi: 10.1186/1471-2229-12-166 (PMC3499179; doi:10.1186/1471-2229-12-166)
Supplement: Additional file 2 — Quantitative real time PCR expression analysis of HvOS2 in the large-seed cultivars, Byzantio, and a small-seed cultivar, Ippolytos, at different stages of seed development. [file 1471-2229-12-166-S2.doc]

**Additional File 2.**

**Quantitative real time PCR expression analysis of *HvOS2* in a large-seed cultivar, Byzantio, and a small-seed cultivar, Ippolytos, at different stages of seed development.**

Expression values were normalized to those of *HvActin*. The relative expression ratio of each sample is compared to the control group which was BIF (Byzantio immature flowers) and set arbitrarily to 1. B, cultivar Byzantio, (black bars); IP, cultivar Ippolytos (grey bars). IF, Immature flower; 1-3, Seed 1-3 DAF; 3-5, Seed 3-5 DAF; 5-10, Seed 5-10 DAF; 10-15, Seed 10-15 DAF; 15-20, Seed 15-20 DAF. Data represent mean values from two independent experiments with standard deviations. Values significantly different (P<0.05) from the control group (BIF) are marked with an asterisk.


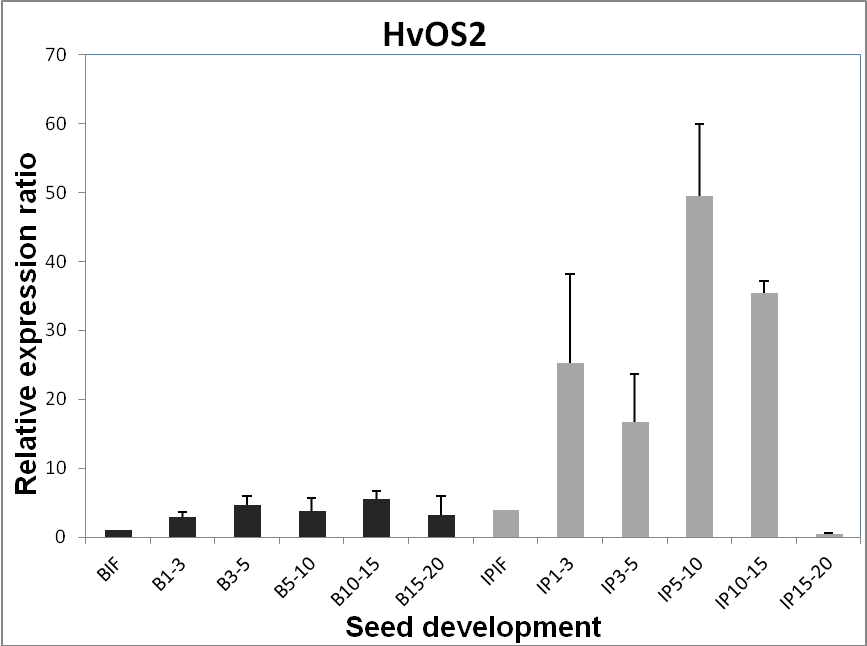


*

*

*

*

*

*
