# Supplementary material for: The study of two barley Type I-like MADS-box genes as potential targets of epigenetic regulation during seed development
Source: BMC Plant Biol. 2012 Sep 17;12:166. doi: 10.1186/1471-2229-12-166 (PMC3499179; doi:10.1186/1471-2229-12-166)
Supplement: Additional file 3 — Detailed exon/intron organization of the HvOS2 , Bradi2g59190, Bradi2g59120, OsMADS65, and ZmB4FML1. [file 1471-2229-12-166-S3.doc]

**Additional File 3.**

**Genomic organization of cereal Type I-like MADS-box genes**

**HvOS2**

**
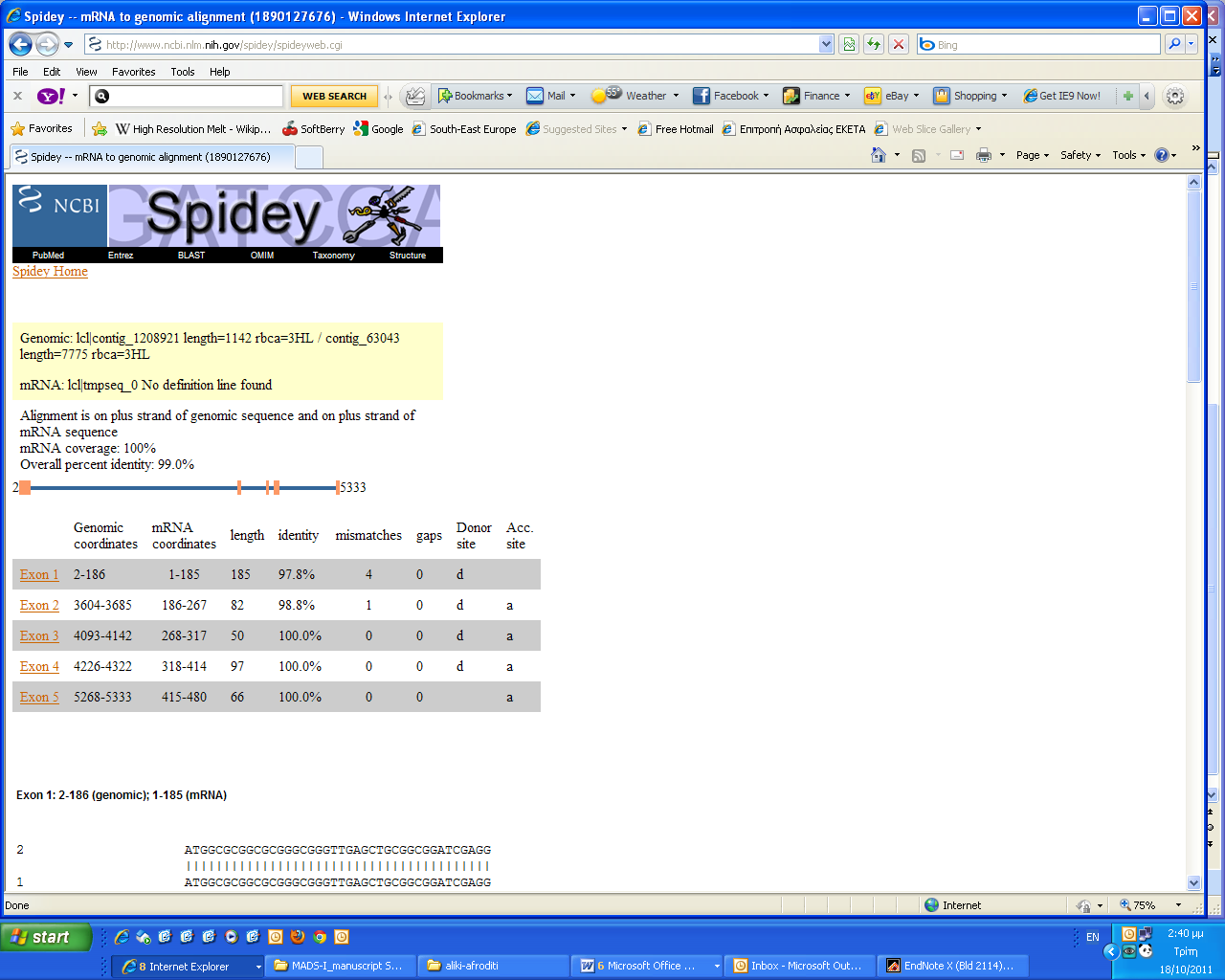
**

**Oryza sativa_OSMADS65_Os1g0922800_Q9XJ61**

Genomic organization:

**Oryza sativa_OSMADS65_Os1g0922800_Q9XJ61**

**
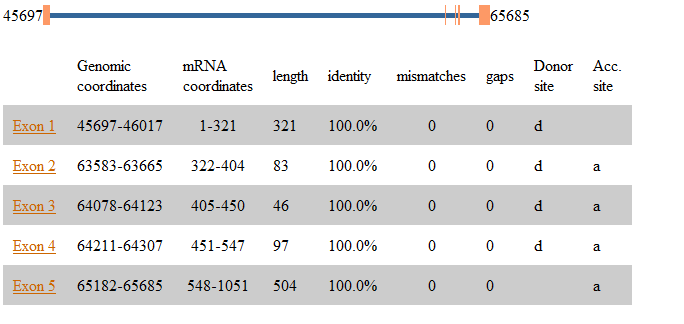
**

**Brachypodium distachyon_Bradi2g59190**


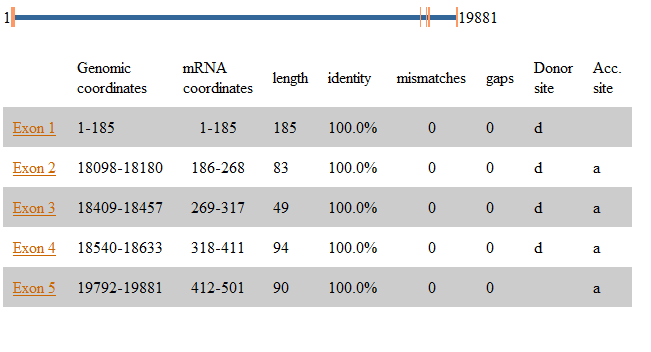


**Brachypodium distachyon_Bradi2g59120**

**
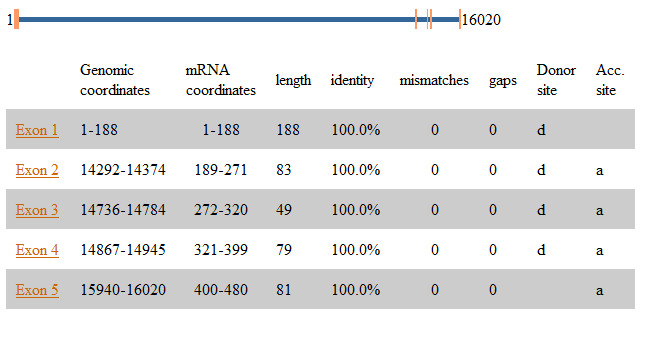
**

**Zea mays_ZmMADS1_GRMZM2G171650_B4FML1**

**
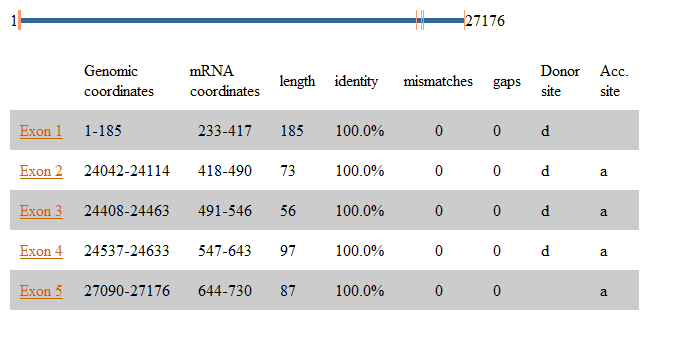
**

**Brachypodium distachyon_Bradi2g59190**
