# Supplementary figures and images for: The study of two barley Type I-like MADS-box genes as potential targets of epigenetic regulation during seed development
Source: BMC Plant Biol. 2012 Sep 17;12:166. doi: 10.1186/1471-2229-12-166 (PMC3499179; doi:10.1186/1471-2229-12-166)

**Additional File 4.**

**Mapping in silico**

POPA3_0092 on chromosome 3 on the barley genetic map

**
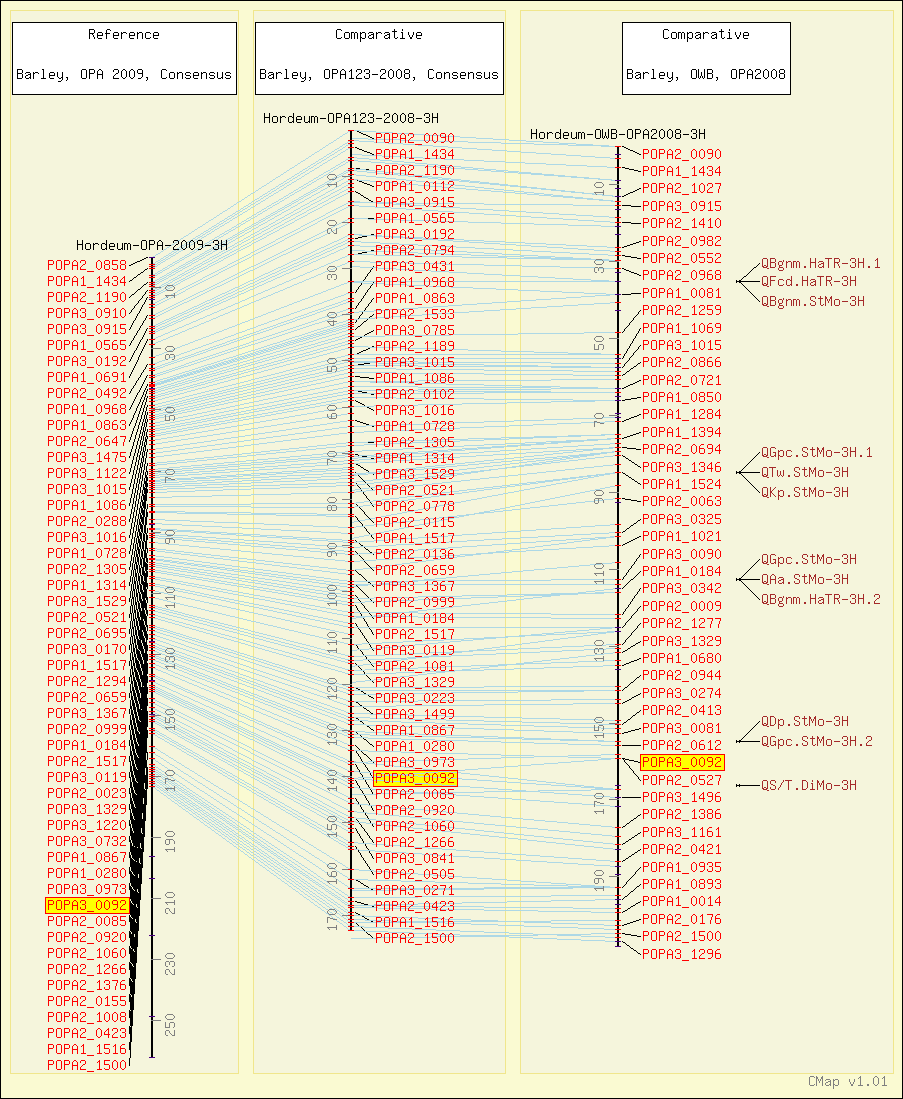
**

Supplement: Additional file 4 — Mapping in silico. [file 1471-2229-12-166-S4.doc]
