# Supplementary material for: The study of two barley Type I-like MADS-box genes as potential targets of epigenetic regulation during seed development
Source: BMC Plant Biol. 2012 Sep 17;12:166. doi: 10.1186/1471-2229-12-166 (PMC3499179; doi:10.1186/1471-2229-12-166)
Supplement: Additional file 5 — Analysis of 5’ upstream regions of HvOS1 and HvOS2. [file 1471-2229-12-166-S5.doc]

**Additional File 5.**

**Analysis of 5’ upstream regions of *HvOS1* and *HvOS2*.**

Regions 5’ upstream from the ATG translation initiation site, as well as coding regions are depicted. Translation initiation codons, ATG, and stop codons TAG and TGA, respectively, are shown in bold boxes. Regions shaded in grey indicate a statistically significant prediction for CpG islands. Cis acting elements are boxed. Cis elements in *HvOS2*: ACGT, ABA responsive element; CGTCA, JA responsive element from barley; TGAGTCA, GCN4- endosperm specific; TATTATAA, TATA box; CAAT, CAAT box. Cis elements in *HvOS1*: GACGTGT, ABA responsive element; TGACG, JA responsive element from barley; GTCAT, Sk1 motif – endosperm specific element; CCTTTTG, Gibberellin responsive element; TAATAT, TATA box.

***HvOS1***

Region 1F

ACTATAGGGCACGCGTGGTCGACGGCCCGGGCTGGTAAAAGTTAAGTTACAAGTTATTCGGATGCCAATGAGCAAATT

JA response

ABA response respons.

TAAGATGTGACGAGTCGTGTTGGACGTGTCCGGATATGTCAACGGACATATGAGGATCCAAATTTACTGTCACGGCTG

TAGATGCTCTTACTTATGCGGCATTTCTAAGCACGAGAGATCCATCCACACAGGCTAAACTTAGCTGCAAATACGCAC

Sk1-endosp.

TATA

GAGTTAGGGGTCGCGACCACAAAATCGTCATCCTCCGGCGGGACTTTCACCTTAATATCCGCGAACCTGGAAACAAGG

JA response

AAAAAAAAAACGGCGACGGCCAATGGCGCTCCAACGGTGGCTCCTGGGCGGGTCCCCTGATTGATTTTTCCCAGGATG

Gibberrellin response

GGAAATTCCTCGCGCAGTCGCCTTCTTTATTTGGCCGGGTCGCTTCCTTTTGTTACCCCTTCGATCCATACCTTGC**C**C

Region 1R.

TACTTACCTTCGCCGCCACCACCACCGACGAGAGGGACTGAGATAGAGAGACGGGCTGCGGCCGGAGGAGGAGGATGG

CGCGGCGCGGGCGGGTTGAGCTGCGGCGGATCGAGGACCGGACGAGCCGGCAGGTGCGCTTCTCCAAGCGCCGCGCGG

GGCTCTTCAAGAAGGCCTTCGAGCTCTCGCTCCTCTGTGACGCCGAGGTCGCGCTGCTCGTCTTCTCCCCCGCCGGCA

AGCTCTACGAGTACTCCTCAGCCAGCATTGAAGGTACATATGACCGGTATCAGCAATTTGCGGTTCCTGGAAGGACCC

TGGATCAAGAAGATGCAACTGTCAGCAATGATGAAGATCCTTCAAATATGCAGTCAAGGCTTAGCGAGATTGCTGCCT

GGTCTCTCGATAATAATGCTGACAACTCAGATGCCAGTAGTTTGGAGAAACTGGAGAAACTACTAAAAGATGCTCTGA

GAATTACAGAATCTAAGAAGACGTTGGCAAAACAAAATAGTGGCGGGAGCACGAGCGAGAGAGCTCCAACGGACCTAG

Region 2F

GGGGCATGAGAATGGGAGGAAGGCTTGAGTAGAGCAAAGTTCCCCTGTGCTGTGTATGTATTTGATACTTACTATATC

TTGCTGCCCCCACACACTGTTGCCCTGTTAGGCTGTAATTGGCCAAGAAATACTTGTCAAGGCTGCTACTTGTAAGGT

Region 2R

GTGAGGGAGGAGGGGCAAAATTTAACGAAAGTGTGGGTTAATACTGATGCATCGGGTCCGTGCTATGAAGAGACCCTG

TATTACCTACGACTATGTGTGTTGCTTGATCTTATTAAAAAAAAAAAAAAAA

***HvOS2***

CTATAGGGCACGCGTGGTCGACGGCCCGGGCTGGTCTGTTGGATCAAAGGCTGGTGGAACATTGGATGCACACATCAATTTAAT

ABA response respons.

TTGAACGTGATTGTAATTACCACGGGATTTGTGATTTGAAGAAACTAATACCGGATTTTAAGTAACTGACCAGTTTCAAGAAAT

GAATTATATACTCTCTAAAAGAACTAATCTAGCGTAGTGGGTATCCGTGTAATGCGTCTTTCTTCCTTCTTAAGGAAAAATAAT

ABA response

TCTCAATGTCCCTTCAAATTGCATCCAACATAAATCAAACGTGTTTCAACCTAAAGAAAATGTGTTCCTAGTTAGCAGTTTTTC

TCCAGCATACCAAATATATGTTTCCGTCGACAGAAATTTGCCTCCGTATATATCAAAAATTGCTTTCATATGCTGAATGGAATT

TTATTTCCATGTTGAAAGAAAATAAATTTGCTTCCATCCTTATTTAGGAATAACAATGCATTCATACATTCACATTAAATGCTT

CTCGTAATTTATATATTTGCTTCAATCAGCCAGAGTGAATCACGATGGCTGCCGCTTTTGTTTATCAAGACAAGTGCGCGGATG

Region 1F

TGGGCTATTACAGCCGCCTCTCATGGCTTTGAATGGACGACCTCTGCTTCCTGCTAGGGATTGGAAAGGCAAAGAGTGCAGAGG

AAGGGGAGGCAGAGGGGCGGCGGAGGGATCCCGAGCATGGGCGAATTATGGATCAGCTTTGAACCGTGATTGCATGATGGGTGA

TGGGCAACTTTCTTCCTTGGTTAAAGGGCGCACAGAGTGAAGGAAGGGAGATGTTCTCAGCCATACGATGGAAACAAATCGATT

GTACATGATTGGTTTGAACTTTGATGAAGGGCAAAAAGACGGATGAGGAAAATTGTTTCCCTTTGTTAAAGACCATACACATTT

ATCTCTATCCTTTTTTCCTCTTTGCTGGTATCTCTCTCGTACCATAGTAACACACAAATCTTATTATTCTTGCCAAGATTTGTC

AAATCGCCTCTCAAGCTCCTTCTAAACCTGGTACTCCCCCACCCTCGTGATTTTTTTCTTTTGCAACAGTAATACTCCCTCCGT

CCCAAAATAATTGTCCTAAGCTTAGAACAATTTTGTATTAGAGCTAGTACATAGTCAAGATAGTtATTTGGACgGAgGAGTATT

AAAGTTTACATTCACATTACACAATCCGGCAATTTTCGTATATAGAGGGGTACTGCCCAGCCAAGAATTGCAACTCACATTCAC

ABA response

ATTAGACCAAAATATAGTACTCGTATCCATTCATTCACGTTTCGATACTCCTACGGTTTTTTCTCTTTATGCGTTTTGCAATCT

TCAAATGGAAGAGGCAGGCACCATGTGTCGTGTGGTTAGCGGTAGAGCCGTTAATCTTGGTAGCAAAATTCCCCTGCTTTCACA

ACCAGTCACCATCTCAACATGAGGAGATGCAGCCAAGGAGGAGGGGCGCGGGCCCATCGCGCTGTCGGGCATGCAAGCGGCCAC

ACAGACAGCAGCCCTTGCCTTTCACGGTATCCTTATCCTTGCGAAATTTAACCACTTTCGGCTCATTTCATTGGTCCTCACCCG

ABA response

TGCCCACACTCGCCACAGGCTGATATTTCGTGTGCCGTACTACGTCCTAGCTGGAGCCCTCCGCTCCCACACACACGTTGTTTT

GCN4-endosp.

TTCACAGCGTGGATAAGGACGGGATGAGTCAGCGCGGAGCTGTGCAGGGGCCGCGCGCCTCGGCTGCGCGTCCCTGTTTATTTG

TATA

Region 1R

AAAAAGGCCGGTGCCAAACCGGAGCTCTCCAGTCTCCTCTATTATAAGCAAAACAATGGGCTCGTGCATTCTCTGGGTCGCCGT

CGACTCCACGTTTTATCTGCTGCTGCACACCAAAGCATGAGTGACTGGTGGAGTGGATCAAGGAAGAAAGCACTGTACCACTAG

CAGTAAGAACTAAGAAGAGAGCTTGCCCAGCGGAAGAAACCGACCAAGGAGCGGCAGAGAATGCGGCCAAGAGCGCGCCGTGTC

JA response

CGAGCTGTACGGTGATCCGTCAGCCCACCCGCCCCTCTCGCCCACTCCCCTGAGCCGGAGGAGCCCCCCCATCTCTGCACTCCC

TGATTCCCTTCCAACGACCGAAAAAAGGAGAAAGAGACGAGCGACGGCCGGAGGAGAAGAAGGAGGCTAGCTTTTCCGGCGGGC

Region 3F

GATGGCGCGGCGCGGGCGGGTTGAGCTGCGGCGGATCGAGGACCGGACGAGCCGGCAGGTGCGCTTCTCCAAGCGCCGCTCGGG

GCTCTTCAAGAAGGCGTTCGAGCTGTCGGTCCTCTGCGACGCCGAGGTCGCGCTGCTCGTCTTCTCCCCCGCCGGCAGGCTCTA

Region 3R

CGAGTACGCCTCCTCCAGCATAGAAGGTACATATGACCGCTATCAGGCATTTGCAGGAGCCGGAAAGGATGTGAGTGAAGGCCG

TGCAAGTAACAACAATGATGGAGATCCTTCAAATATACAGTCAAGGCTTAAAGATATTACTTCCTGGTCTCTTCAAAACAATGC

TGATGACTCAGATGCTAATGAACTGGTGAAACTGGAGAAACTATTGACAGATGCTTTGAAGAAGACAAAATCCAAGAAGATAT

Region 4R

Region 4F

TGGCGCAACGAAATAGCGGTGCGGGCACGATTGCGAGTGGCGAGAACTCCAGAAGGTTTTGAGCAGCCATGTGATGTGCATGCA

Region 2F

TTTGGTACAAGCAAACCTCTGTTGTTGATGCCACACTCTTCCCCAGTTATTGGGTATGAAAGAGTTGTGAAGGCTGCTGCTTGC

GAGGTGCAGCAAAGGCATGAGGGGCAAATTCAATAAAAGGGTGGTGGGTTAACTCTTAACAGCAACAGTGCGTGTGTCCTTGTT

Region 2R

ATCTGTTGCCTTACCTTTTGTGCTTTCCTAGGGCTGTGTGTGATGCTCATATTAACATATCAGTCTGCTGTCAGTCTTCACTAC

ACAGGTTATACGCAACAGCGGCCGCTGCCAAACATAGATCAATGGGTTCAGCTCGAAGAAAAACACTGAACAAATGAATTTCCT

GGACCATGTACACATGAATACCAACATGTGACACGGTACGATGATCCCAGCTTGCATAATTACAGTTGCGAGATAGCTGGTTAA

CGACTGATAAGACCC
